# Effect of unaffordable medical need on distress level of family member: analyses of 1997–2013 United States National Health Interview Surveys

**DOI:** 10.1186/s12888-017-1483-z

**Published:** 2017-09-02

**Authors:** Hui Jun Chih, Wenbin Liang

**Affiliations:** 1 School of Public Health, Faculty of Health Sciences, Curtin University, GPO Box U1987, Perth, WA 6845 Australia; 2 National Drug Research Institute, Faculty of Health Sciences, Curtin University, GPO Box U1987, Perth, WA 6845 Australia

**Keywords:** Health care and services, Cost, Mental health, Public health

## Abstract

**Background:**

Reduced funding to public health care systems during economic downturns is a common phenomenon around the world. The effect of health care cost on family members of the patients has not been established. This paper aims to explore the relationship between affordability of health care and vulnerability of family members to distress levels.

**Methods:**

Data of a total of 262,843 participants were obtained from 17 waves (1997–2013) of the United States National Health Interview Survey. Multinomial logistic regression was used to investigate psychological distress level as a result of having family members who experienced unmet medical needs due to cost.

**Results:**

Among participants without family members who experienced unmet needs for medical care due to cost, risks of having ‘moderate’ (score of 5–12) or ‘serious’ (score of 13 or above) level of psychological distress were 1.0% and 11.5%, respectively. Risks of having ‘moderate’ or ‘serious’ level of psychological distress were 3.1% and 23.4%, respectively among participants with family members who experienced unmet needs. The adjusted relative risk ratio of ‘moderate’ and ‘serious’, as compared to ‘normal’ level of psychological distress, were 1.58 (95% confidence interval: 1.47–1.69) and 2.09 (95% confidence interval: 1.78–2.45) if one’s family members experienced unmet medical needs.

**Conclusions:**

Unmet medical needs due to cost increases risk of distress levels experienced by family members. Careful planning and adequate funding to public health care system could be implemented to prevent any unnecessary detrimental effect on mental health among family members of the unwell and any further increment of the prevalence of mental illnesses. This recommendation aligns with the World Health Organization Mental Health Action Plan 2013–2020.

## Background

Health care systems in many developed countries are funded from public sources to provide equitable access to all based on needs [1]. Despite the idealism in providing equitable access to health care for all, access was not equally distributed across income groups [1]. Increased cost-sharing or more out-of-pocket expenses by patients during economic downturns or reduced health care funding reduced access to health care for some and subsequently increased their susceptibility to some infectious diseases, worsened their conditions of chronic diseases and increased mortality rates [2–4].

While poor access to health care can lead to negative impact on physical health of patients, the cost to access health care may also add to one’s family financial burden [5]. The financial burden can negatively impact the family members, who may already be under stress when providing daily care to their ill relatives [6–8]. Their mental health may be sub-optimal and yet overlooked. Meanwhile, the World Health Organization (WHO) has highlighted the importance of reducing prevalence of mental illness in its Mental Health Action Plan 2013–2020 [9].

A search through the PubMed, Science Direct and Web of Science databases using the following keywords: “unmet medical needs” AND “mental distress” AND “family member” AND “cost” from 1st January 2010 to 1st January 2017 yielded 426 articles. However, after a closer examination, there is no publication on this topic. It is therefore paramount to investigate the impact of unmet medical care and the vulnerability of family members to psychological distress. The aim of this study was to investigate the effect of having a family member with unaffordable medical cost on one’s psychological distress level.

## Methods

### Study design and setting

Data from 17 waves (1997–2013) of National Health Interview Survey (NHIS) were collated from Integrated Health Interview Series of United States National Health Interview Survey: Minnesota Population Center and State Health Access Data Assistance Center, Integrated Health Interview Series: Version 5.0. Minneapolis: University of Minnesota, 2012 (http://www.ihis.us). The NHIS was conducted by the National Center for Health Statistics (NCHS), Centers for Disease Control and Prevention to provide estimations of health indictors at national level [10–12].

### Participants

Multistage probability sampling technique was applied to select representative sample of 100,000 people of the wider population (40,000 households) every year and weighted for key demographic variables to assure representativeness of the sample (https://www.ihis.us/ihis/userNotes_sampledesign.shtml).

### Measures

Details of the survey sampling strategy and data collection methods were described elsewhere [10, 12]. Briefly, for this study, one adult aged 18 years or older was selected per family to provide detailed information regarding his/her demographics, access to medical care service, physical and mental health, which included an assessment on nonspecific distress level in the past 30 days using the standard Kessler’s 6 (K6) questionnaire [13]. The six questions asked were: “During the past month, how much of the time did you feel … so sad nothing could cheer you up?”; “… nervous?”; “… restless or fidgety?”; “… hopeless?”; “… that everything was an effort?”; and “… worthless?”. Each of the question was scored with zero for “None of the time”, one for “A little of the time”, two for “Some of the time” and three for “Most of the time” and four for “All of the time”, yielding a range from zero to 24. A K6 score of 5–12 was used to indicate presence of ‘moderate’ mental distress while score of 13–24 was used to indicate presence of ‘serious’ mental distress [14]. The questionnaire has good validity and reliability [15] and the cut-off points are clinically validated [14]. In this study, unmet needs of medical care was defined as answering “yes” to the question: “whether during the past 12 months, was there any time when needed medical care, but did not get it because couldn’t afford it”.

### Statistical analyses

The information obtained from the NCHS was anonymized and de-identified prior to analyses. The effect of having family members who had unmet needs for medical care on the risk of having i) moderate or serious (K6 score ≥ 5) psychological distress level and ii) serious (K6 score 13–24) psychological distress level in the past 30 days were first assessed in two multivariate Poisson regression models. Psychological distress level was further classified into three categories based on their K6 score: 0–4 (normal), 5–12 (moderate) and 13–24 (serious). Multinomial logistic regression was then used to investigate the effect of having family members who had unmet needs for medical care on their psychological distress level. In order to control for the potential confounding effect of participant’s health on his/her distress level, analyses were restricted to participants who: i) were living with two or more members in a single family household; ii) rated their health status as good, very good or excellent; and iii) were free from any limitation due to physical or mental problems, without unmet needs of medical care. Potential confounding factors such as age, gender, race, marital status, highest educational attainment and health status, whether having any family members with activity limitations, family income comparing to poverty threshold and year of survey were controlled for in the multivariate models. Participants with missing information on these potential confounding factors were excluded in the multivariate analysis. Data from a total of 262,843 participants were included. Sampling weight was applied in all analyses [10]. Significance level of 0.05 was applied.

## Results

The risks of having moderate and serious (K6 score ≥ 5) or serious (K6 score ≥ 13) level of psychological distress were 23.4% and 3.1%, respectively among participants with family members who had unmet needs for medical care. The risks among participants without family members had unmet needs were significantly lower at 11.5% and 1.0%, respectively. In other words, having family members with unmet needs for medical care were associated with about 100% and 200% increase in the risk of having at least moderate and serious level of psychological distress, respectively.

Overall the multivariate analyses showed that the risk of moderate or serious and serious psychological distress level were significantly higher (50% and 80%, respectively) among participants who had family members with unmet medical needs (Tables 1 and 2). Estimates from both the multivariate Poisson regression models and multinomial logistic regressions (Table 3) are consistent with the findings from univariate analysis albeit the effects become smaller after controlling for confounding variables.

**Table 1:** Relative risk (RR) of moderate and serious psychological distress level^a^ in the past month

		RR	95% confidence interval of RR	
Any family member with unmet need for care	No	1.00		
	Yes	1.45	1.38	1.52
Any family member with activity limitation	No	1.00		
	Yes	1.32	1.27	1.36
Age	18–24	1.00		
	25–34	0.92	0.88	0.96
	35–44	0.85	0.81	0.89
	45–54	0.76	0.72	0.80
	55–64	0.58	0.54	0.61
	65–74	0.43	0.40	0.47
	75+	0.38	0.34	0.43
Gender	Male	1.00		
	Female	1.29	1.26	1.33
Race	White	1.00		
	Black	0.85	0.82	0.89
	Other	0.90	0.86	0.95
Marital status	Married, spouse present	1.00		
	Married, spouse absent	1.31	1.13	1.51
	Separated	1.59	1.48	1.70
	Divorced	1.35	1.28	1.42
	Widowed	1.65	1.50	1.81
	Living with partner	1.28	1.22	1.34
	Never married	1.23	1.17	1.28
Education level	Never attended/kindergarten only	0.99	0.80	1.23
	Grade 1, 2, 3, or 4	0.91	0.79	1.04
	Grade 5, 6, or 7	0.86	0.79	0.94
	Grade 8	1.03	0.92	1.15
	Grade 9, 10, or 11	1.10	1.05	1.16
	Grade 12	0.99	0.96	1.03
	1 to 3 years of college	1.00		
	4 years college/Bachelor’s degree	0.83	0.80	0.87
	5+ years of college	0.81	0.76	0.85
Health Status	Excellent	1.00		
	Very Good	1.38	1.33	1.42
	Good	1.88	1.82	1.95
Ratio of family income to poverty threshold	< 1.00	1.22	1.16	1.28
	1.00–1.99	1.16	1.11	1.21
	2.00–3.99	1.09	1.06	1.13
	4.00+	1.00		
Year of survey	1997	1.00		
	1998	0.96	0.90	1.02
	1999	0.82	0.77	0.88
	2000	0.84	0.79	0.90
	2001	1.08	1.02	1.15
	2002	0.85	0.80	0.91
	2003	0.90	0.84	0.96
	2004	0.93	0.87	0.99
	2005	0.91	0.85	0.97
	2006	0.82	0.76	0.89
	2007	0.74	0.68	0.80
	2008	0.89	0.83	0.96
	2009	0.93	0.87	1.00
	2010	0.93	0.87	0.99
	2011	0.77	0.72	0.83
	2012	0.72	0.67	0.77
	2013	1.01	0.95	1.08

^a^ *K6 score ≥ 5*

**Table 2:** Relative risk (RR) of serious psychological distress level^a^ in the past month

		RR	95% confidence interval of RR	
Any family member with unmet need for care	No	1.00		
	Yes	1.84	1.58	2.15
Any family member with activity limitation	No	1.00		
	Yes	1.5	1.38	1.73
Age	18–24	1.00		
	25–34	0.96	0.82	1.12
	35–44	0.90	0.76	1.06
	45–54	0.82	0.68	0.98
	55–64	0.56	0.45	0.70
	65–74	0.45	0.34	0.60
	75+	0.32	0.22	0.46
Gender	Male	1.00		
	Female	1.58	1.43	1.75
Race	White	1.00		
	Black	0.86	0.75	0.99
	Other	1.01	0.84	1.20
Marital status	Married, spouse present	1.00		
	Married, spouse absent	1.61	1.10	2.36
	Separated	2.16	1.76	2.65
	Divorced	1.63	1.38	1.92
	Widowed	2.26	1.69	3.02
	Living with partner	1.34	1.14	1.57
	Never married	1.26	1.08	1.47
Education level	Never attended/kindergarten only	1.66	0.84	3.28
	Grade 1, 2, 3, or 4	1.60	1.14	2.24
	Grade 5, 6, or 7	1.20	0.94	1.54
	Grade 8	1.72	1.24	2.39
	Grade 9, 10, or 11	1.53	1.31	1.79
	Grade 12	1.12	1.00	1.26
	1 to 3 years of college	1.00		
	4 years college/Bachelor’s degree	0.62	0.52	0.74
	5+ years of college	0.53	0.41	0.67
Health Status	Excellent	1.00		
	Very Good	1.26	1.12	1.43
	Good	2.09	1.85	2.36
Ratio of family income to poverty threshold	< 1.00	1.87	1.58	2.21
	1.00–1.99	1.52	1.31	1.77
	2.00–3.99	1.23	1.07	1.40
	4.00+	1.00		
Year of survey	1997	1.00		
	1998	0.82	0.66	1.01
	1999	0.68	0.54	0.86
	2000	0.94	0.76	1.17
	2001	0.88	0.72	1.09
	2002	0.98	0.78	1.22
	2003	0.92	0.74	1.14
	2004	0.76	0.59	0.97
	2005	0.69	0.55	0.87
	2006	0.76	0.59	0.98
	2007	0.67	0.51	0.89
	2008	0.68	0.53	0.88
	2009	0.75	0.58	0.97
	2010	0.80	0.64	1.01
	2011	0.66	0.52	0.84
	2012	0.64	0.49	0.82
	2013	1.01	0.81	1.26

^a^ *K6 score ≥ 13*

**Table 3:** Relative risk ratio (RRR) of moderate^a^ and of serious^b^ psychological distress levels in the past month

		‘Moderate’ psychological distress level			‘Very high’ psychological distress level		
		Adjusted RRR^c^	95% confidence interval of adjusted RRR		Adjusted RRR^c^	95% confidence interval of adjusted RRR	
Any family member with unmet need for care	No	1.00			1.00		
	Yes	1.58	1.47	1.69	2.09	1.78	2.45
Any family member with activity limitation	No	1.00			1.00		
	Yes	1.38	1.32	1.44	1.66	1.47	1.87
Age	18–24	1.00			1.00		
	25–34	0.90	0.85	0.95	0.94	0.80	1.10
	35–44	0.82	0.77	0.87	0.86	0.73	1.02
	45–54	0.71	0.67	0.77	0.76	0.63	0.92
	55–64	0.53	0.49	0.57	0.50	0.40	0.63
	65–74	0.38	0.34	0.42	0.39	0.29	0.52
	75+	0.33	0.29	0.38	0.27	0.18	0.39
Gender	Male	1.00			1.00		
	Female	1.33	1.28	1.37	1.67	1.51	1.85
Race	White	1.00			1.00		
	Black	0.82	0.78	0.87	0.83	0.72	0.96
	Other	0.87	0.82	0.93	0.98	0.82	1.18
Marital status	Married, spouse present	1.00			1.00		
	Married, spouse absent	1.34	1.56	1.89	1.71	2.01	3.08
	Separated	1.72	1.31	1.49	2.49	1.46	2.07
	Divorced	1.40	1.52	1.94	1.74	1.84	3.38
	Widowed	1.72	1.26	1.41	2.49	1.20	1.66
	Living with partner	1.34	1.20	1.35	1.41	1.12	1.54
	Never married	1.27	1.11	1.63	1.31	1.15	2.54
Education level	Never attended/ kindergarten only	0.91	0.70	1.20	1.65	0.81	3.35
	Grade 1, 2, 3, or 4	0.81	0.67	0.97	1.55	1.09	2.21
	Grade 5, 6, or 7	0.80	0.71	0.89	1.16	0.90	1.49
	Grade 8	0.96	0.83	1.11	1.73	1.23	2.43
	Grade 9, 10, or 11	1.09	1.02	1.17	1.58	1.34	1.86
	Grade 12	0.98	0.94	1.02	1.12	0.99	1.26
	1 to 3 years of college	1.00			1.00		
	4 years college/ Bachelor’s degree	0.83	0.78	0.87	0.60	0.50	0.73
	5+ years of college	0.81	0.76	0.87	0.52	0.40	0.66
Health Status	Excellent	1.00			1.00		
	Very Good	1.46	1.40	1.52	1.33	1.17	1.51
	Good	2.10	2.01	2.19	2.39	2.11	2.70
Ratio of family income to poverty threshold	< 1.00	1.22	1.14	1.30	1.96	1.65	2.33
	1.00–1.99	1.16	1.10	1.22	1.56	1.34	1.81
	2.00–3.99	1.09	1.05	1.14	1.23	1.08	1.41
	4.00+	1.00			1.00		
Year of survey	1997	1.00			1.00	0.65	1.01
	1998	0.97	0.89	1.05	0.81	0.51	0.83
	1999	0.80	0.74	0.88	0.65	0.72	1.14
	2000	0.80	0.74	0.87	0.91	0.72	1.12
	2001	1.12	1.04	1.21	0.90	0.75	1.19
	2002	0.81	0.75	0.89	0.94	0.71	1.12
	2003	0.88	0.81	0.96	0.89	0.58	0.96
	2004	0.93	0.86	1.01	0.74	0.53	0.85
	2005	0.91	0.84	0.99	0.67	0.56	0.95
	2006	0.80	0.72	0.88	0.73	0.48	0.84
	2007	0.70	0.64	0.78	0.63	0.51	0.86
	2008	0.89	0.81	0.98	0.66	0.57	0.96
	2009	0.93	0.86	1.02	0.74	0.62	0.99
	2010	0.93	0.85	1.01	0.78	0.49	0.80
	2011	0.75	0.69	0.81	0.62	0.46	0.77
	2012	0.69	0.63	0.75	0.59	0.81	1.27
	2013	1.02	0.94	1.10	1.02	0.65	1.01

^a^ *K6 score of 5–12*

^*b*^ *K6 score ≥ 13*

^c^ Adjusted for age, gender, race, marital status, highest educational attainment and health status of the main participants, whether having any family members with activity limitations, family income comparing to poverty threshold and year of survey

## Discussion

In the past, reduction or removal of health care funding in some countries contributed to poorer patients’ health outcomes as they could not afford medical care due to cost. This study provides a new insight by directly measuring the relationship between unmet medical care, specifically due to cost, and the vulnerability of family members (not just the main care-giver) to psychological distress. In this study, it was found that health care cost, when linked to unmet medical needs, affected psychological distress level of family members. Specifically, family members of those with unmet medical needs due to cost are at a significantly higher risk of suffering from ‘moderate’ and ‘serious’ level of psychological distress, as assessed by the K6 scale. The risk is comparable to those with a history of epilepsy [16].

A person may experience high psychological distress when they have to make decision when there are conflicts in the choice, and has to ‘trade-off between attributes’ [17]. Family members of patients with unmet medical needs may have to go through ‘distressed financing’ [18] to provide for the unaffordable treatment while sacrificing other needs. Perceived likelihood of negative consequences increases conflicts during their decision making, which can lead to perceived difficult circumstance and increased distress level [17]. It should also be noted that unsecured debt is strongly associated with mental health issues including depression, substance use and suicide [19].

Adequate resources can maintain if not prevent ill health outcome [20]. Effective preventative approaches such as implementing mental health first aid program at rural or urban communities, including but not limited to workplaces or learning environment for general public, teachers and future health professionals [21–24] can be applied in various settings as an early preventative strategy. Systematic documentation of medical and administration costs incurred at various stages of the treatment at various departments and by health professionals as well as barriers to effective care plan can allow better management of ‘cost for value’ for the patients [25, 26]. Quality tools and resources to support organizational improvement integral to high-quality primary care such as coordinated home care program [27, 28] may also be adopted in and out of hospital. Effectiveness of these services in preventing mental illnesses can also be tested in communities in order to provide evidence-based recommendation to decision makers and funding bodies. As part of the planning, it is important to assure that the services are easily accessible as they can mitigate the risk of mental illness of the vulnerable group [29].

The detrimental impact on the mental health of these family members is not exclusive to those living in the United States of America (USA) but everyone who has to undergo difficult decision making process, particularly when the demand of illness is not static [5] and there are dynamic economic changes. The insured ones are not exempted as there are still co-payments and other out-of-pockets expenses [4, 30], which can continue to add to the burden. Due to the high prevalence and disease burden of mental disorders, it is important for clinicians and world leaders to note these preventable consequences and minimize the risk, as in alignment with the WHO Mental Health Action Plan 2013–2020 [9].

This study used a large representative sample of the USA population. The conclusion is thus representative of the wider USA population because it provided a more accurate estimate of the risk of having psychological distress due to unmet medical needs of family members. Due to the nature of cross-sectional design, long term impact on the family members’ distress level is not assessable in this study, particularly when the K6 scale only measures the psychological distress level over a 30 day period. Nevertheless, it is possible that the family members would continue to suffer from psychological distress as their loved ones have unmet medical needs [30]. This further warrants the need to assure adequate provision of resources and preventative health care for the public.

## Conclusions

It is highlighted from this study that unmet medical needs can affect psychological distress of family members, not just the patients themselves. Specifically, family members of those with unmet medical needs due to cost are at a significantly higher risk of ‘moderate’ and ‘serious’ level of psychological distress. These important findings need to be taken into consideration while shaping the funding for our health care systems in order to avoid detrimental effect on mental health of the otherwise healthy family members and to prevent further rise in the prevalence of mental illnesses. This recommendation aligns with the WHO Mental Health Action Plan 2013–2020.

## Abbreviations

K6: Kessler’s 6
NCHS: National Center for Health Statistics
NHIS: National Health Interview Survey
RR: Relative risk
RR: Relative risk ratio
USA: United States of America
WHO: World Health Organization
